# Neutrophil recruitment inhibitory factor: a possible candidate for a novel cytokine

**DOI:** 10.1155/S0962935192000103

**Published:** 1992

**Authors:** W. M. S. C. Tamashiro, B. M. Tavares-Murta, F. Q. Cunha, M. C. Roque-Barreira, R. M. D. Nogueira, S. H. Ferreira

**Affiliations:** 1 Department of Pharmacology Faculty of Medicine of Ribeirão Preto USP Ribeirão Preto SP 14049 Brazil; 2 Department of Parasitology, Microbiology and Immunology Faculty of Medicine of Ribeirão Preto USP Ribeirão Preto SP 14049 Brazil

## Abstract

Inhibitory effect upon neutrophil migration to the inflammatory focus was previously detected in the cell-free incubation fluid of lipopolysaccharide (LPS)-stimulated macrophage monolayers. In the present study we showed that the neutrophil recruitment inhibitory activity from this supernatant was mainly detected in a fraction (P2) obtained by gel filtration chromatography on Sephacryl S-300. P2 fraction was able to inhibit ‘in vivo’ neutrophil emigration induced by different inflammatory stimuli, but it did not affect ‘in vitro’ neutrophil chemotaxis induced by FMLP. When injected intravenously, P2 inhibited oedema induced by carrageenin or immunological stimulus but not the oedema induced by dextran, thus affecting cell-dependent inflammatory responses. It was observed that P2 also induced neutrophil migration when injected locally in peritoneal cavities. This activity was significantly reduced by pretreatment of the animals with dexamethasone. Cytokines, such as IL-8 and TNF-α that are known to exhibit inhibitory effect upon neutrophil migration, were not detected in P2 fraction by highly sensitive assays. Overall the results suggest the existence of a novel cytokine exhibiting ‘in vivo’ neutrophil inhibitory activity, referred as NRIF.


Mediators of Inflammation 1, 49–54 (1992)

### Neutrophil recruitment inhibitory factor: a possible candidate for a novel cytokine

W. M. S. C. Tamashiro, B. M. Tavares-Murta, F. Q. Cunha, M. C. Roque-Barreira,[1] R. M. D. Nogueira and S. H. Ferreira[CA]

Department of Pharmacology,[1] Department of Parasitology, Microbiology and Immunology, Faculty of Medicine of Ribeirão Preto, USP, 14049 Ribeirão Preto, SP, Brazil

[CA] Corresponding Author

**INHIBITORY effect upon neutrophil migration to the inflammatory focus was previously detected in the cell-free incubation fluid of lipopolysaccharide (LPS)-stimulated macrophage monolayers. In the present study we showed that the neutrophil recruitment inhibitory activity from this supernatant was mainly detected in a fraction (P2) obtained by gel filtration chromatography on Sephacryl S-300. P2 fraction was able to inhibit '*in vivo*' neutrophil emigration induced by different inflammatory stimuli, but it did not affect '*in vitro*' neutrophil chemotaxis induced by FMLP. When injected intravenously, P2 inhibited oedema induced by carrageenin or immunological stimulus but not the oedema induced by dextran, thus affecting cell-dependent inflammatory responses. It was observed that P2 also induced neutrophil migration when injected locally in peritoneal cavities. This activity was significantly reduced by pretreatment of the animals with dexamethasone. Cytokines, such as IL-8 and TNF-α that are known to exhibit inhibitory effect upon neutrophil migration, were not detected in P2 fraction by highly sensitive assays. Overall the results suggest the existence of a novel cytokine exhibiting '*in vivo*' neutrophil inhibitory activity, referred as NRIF.**

**Key words:** Cytokines, Macrophage-derived neutrophil recruitment inhibitory factor, Neutrophil migration

### Introduction

Cytokines, such as IL-6, IL-8 and TNF-α, besides their pro-inflammatory effects[1–4] also exhibit anti-inflammatory activities, such as inhibition of neutrophil adhesion to activated endothelial cells[5] and '*in vivo*' inhibition of neutrophil migration into inflammatory focus.[6–8] These cytokines are released by various types of activated cells, such as those of phagocyte mononuclear system,[9,10] polymorphonuclear leucocytes,[11] endothelial cells[5] and T-lymphocytes.[12,13]

In our laboratory we have examined the activity of supernatants from macrophage monolayers stimulated with LPS. It was found that this crude supernatant is able to induce neutrophil migration when injected into peritoneal cavities of dexamethasone-pretreated rats. This activity was referred as macrophage-derived neutrophil chemotactic factor (MNCF).[14] In contrast, neutrophil migration to peritoneal cavities induced by cytokines such as TNF-α and β, IL-1α and β,[15] gamma interferon[16] and IL-8[17] are inhibited by dexamethasone pretreatment. In addition, the same supernatant when intravenously (i.v.) injected was shown to inhibit in a dose dependent manner neutrophil migration to an inflamed site.[18] The factor responsible for this activity was named neutrophil recruitment inhibitory factor (NRIF).

Therefore the objective of this study was to characterize some biological properties of NRIF. For this purpose, we have submitted crude macrophage supernatant to gel filtration chromatography obtaining a fraction (P2) that retained the original inhibitory activity. We also observed that the i.v. administration of P2 was able to block cell dependent oedema. As IL-8 and TNF, cytokines for which neutrophil recruitment inhibitory activity has been described,[6,7] were not detected in P2 fraction, that in addition was eluted far from the elution volume expected for these cytokines, we suggest that NRIF may constitute a novel cytokine.

### Material and Methods

*Animals:* Adult male Wistar rats weighing 150–180 g were housed in a temperature-controlled room and received water and food *ad libitum* until use.

*Immunization procedure:* Animals were actively immunized with 200 μg ovalbumin (OVA) emulsified in 50% complete Freund adjuvant (CFA) (DIFCO, Detroit, MI, USA), by subcutaneous (s.c.) injection. Controls received s.c. injections of CFA. Rats were used in oedema paw assays on 28th day after immunization.

*Preparation of macrophage supernatant:* The method for obtaining NRIF by stimulating macrophage mono-

© 1992 Rapid Communications of Oxford Ltd layers has been described elsewhere.[18] Briefly, the peritoneal cavities of rats were stimulated by injection of thioglycollate medium (DIFCO) (10 ml of 3% thioglycollate medium/rat). Four days later, the peritoneal cells were harvested with RPMI 1640 (Flow Laboratories, McLean, VG, USA) and allowed to adhere in plastic tissue culture dishes for 1 h at 37°C, in an atmosphere of air with 5% of $CO_2$. The monolayers were then washed three times with phosphate buffered saline (PBS, pH 7.4) and incubated for 30 min at 37°C with 10 $\mu g\ ml^{-1}$ of lipopolysaccharide of *E. coli* (LPS, 0111:B4, DIFCO). The supernatants were discarded and the cells (95% macrophages) washed another three more times with PBS, followed by a final 1 h incubation with 5 ml of RPMI medium per dish, at 37°C. The cell-free incubation fluids were subsequently ultradiafiltered through an YM 10 membrane (Amicon Corp, Lexington, MA, USA). The final volume (corresponding to 5% of the original volume) was filtered on 0.22 $\mu m$ membrane (Millipore, Bedford, MA, USA).

*Chromatographic procedure:* One millilitre of macrophage supernatants prepared as described above, containing the material released by $2.5 \times 10^8$ adherent cells, were dialysed against 0.5 M NaCl solution buffered with 0.02 M phosphate pH 7.4, and applied on Sephacryl S-300 column (2.6 × 70 cm; Pharmacia, Uppsala, Sweden) at a flow rate of 8 $ml\ h^{-1}$. The S-300 column was previously calibrated with a molecular weight standards kit (Pierce Chemical Co., Rockford, IL, USA) containing: ferritin (540 KDa), catalase (240 KDa), aldolase (158 KDa), BSA (67 KDa), OVA (43 KDa), chemotrypsinogen (25 KDa) and cytochrome c (12.4 KDa). Blue dextran 2 000 (Pharmacia) was used to determine the void volume of the column. Absorbance at 280 nm was determined for each fraction collected (2 ml fractions). After desalting on a PD10 column (Pharmacia), the fractions were pooled (Pool 1–Pool 10; three fractions/pool), starting with the fraction corresponding to the void volume of the column (fraction 23), and assayed for neutrophil migration activities as described below.

*In vitro neutrophil migration assay:*

*Preparation of human neutrophils.* Viable and highly purified preparation of human neutrophils were obtained from heparinized venous blood of healthy subjects by mono-poly-resolving medium (Flow Laboratories, Irvine, Scotland) fractionation, according to manufacturer's instructions. Isolated neutrophils were washed three times with RPMI 1640 medium and then resuspended in RPMI medium containing 0.1% bovine serum albumin (Sigma, St. Louis, MO, USA) (RPMI-BSA).

*Pretreatment of neutrophils.* In order to determine if S-300 chromatographic fractions presented '*in vitro*' inhibitory activity, neutrophil suspensions ($10^6$ cells/ml) were incubated for 30 min at 37°C, in a humidified incubator with 5% $CO_2$, either in the absence or in the presence of the pooled chromatographic fractions of macrophage supernatant, now referred as fractions P1 to P10, prior to testing for chemotactic response to *N*-formyl-methionyl-L-leucyl-L-phenylalanine (FMLP, Sigma) in microchemotaxis assay. The chromatographic fractions were diluted in RPMI-BSA in order to give solutions containing the equivalent to the product released by $10^7$ adherent macrophages/ml and were present throughout the assay.

*Chemotaxis assay.* Chemotaxis was performed in 48-well chemotaxis chambers (Neuroprobe Inc., Cabin John, MD, USA). Polyvinylpirrolydone free polycarbonate filters (0.5 $\mu m$) (Millipore) were placed between the upper and lower chambers. Twenty-five $\mu l$ samples of either chemoattractant or RPMI-BSA were added to the wells of the lower chamber. FMLP was used at concentration of $5 \times 10^{-8}$ M in RPMI-BSA. Fifty $\mu l$ samples of pretreated neutrophils were added to the wells of the upper chamber. The chemotaxis chambers were incubated for 1 h at 37°C in 5% $CO_2$. The cells which have migrated to the lower side of the filter were fixed and stained with Diff-Quick Stain Kit (American Scientific Products, McGraw Park, IL). Neutrophils were counted by using a 100 × objective in at least five random fields. Experiments were performed in triplicate for each variable, and the means determined. The results were expressed as number of neutrophils per field. In each experiment, untreated neutrophils migrating toward FMLP were used as positive reference.

*In vivo neutrophil migration assay:*

*Inhibition assay.* Inhibition assays were performed as described elsewhere.[18] Briefly, S-300 chromatographic fractions (P1–P10 fractions) or different amounts (3 pg–3 $\mu g$) of mouse recombinant TNF-$\alpha$ (mrTNF-$\alpha$), which was a gift from Dr. M. A. Palladino Jr. of the Cell Biology Department, Genentech Inc., South San Francisco, CA, USA, were i.v. injected into penial venous sinus of the rats. Thirty min later, the animals received an intraperitoneal (i.p.) injection of carrageenin (300 $\mu g$/3 ml) (Marine Colloids, Inc., USA), FMLP solution ($10^{-7}$ M/3 ml) or LPS (5 ng/3 ml). Controls received i.v. injections of PBS. Four hours after i.p. injections, the rats were sacrificed in a $CO_2$ chamber, their peritoneal cavities were washed with 10 ml of PBS containing 5 IU $ml^{-1}$ of heparin and total and differential cell counts in the lavage fluid were performed as described elsewhere.[19] In subsequent biological assays, 0.2 ml (equivalent to the product released by $5 \times 10^6$ cells) of the fraction retaining maximal inhibitory activity was i.v. injected per animal, except when indicated in the Results section. The results are expressed as means $\pm$ SEM of the number of neutrophils per ml of peritoneal wash.

*Detection of MNCF activity.* The S-300 chromatographic fractions were also assayed for the presence of MNCF activity as described elsewhere.[14] Briefly, the fractions were injected into peritoneal cavities (3 ml/cavity, equivalent to the material released by $5 \times 10^6$ macrophages) of dexamethasone-pretreated rats (0.5 mg $kg^{-1}$, s.c., 1 h before). Controls received sterile saline both s.c. and i.p. injected. After 4 h, neutrophil migration was measured as indicated for inhibition assay.

*TNF activity assay:* TNF content in macrophage supernatants and S-300 chromatographic fractions was measured by using a highly TNF-sensitive cell line, WEHI 164 clone 13, as described elsewhere.[20] WEHI 164 clone 13 was a gift from Dr. M. A. Palladino Jr. (Genentech Inc, USA). Briefly, WEHI cells were seeded in microplates (Costar 3596, Cambridge, MA, USA) at concentrations of $4 \times 10^4$ cells/well in 50 $\mu$l of growth medium (RPMI 1640 medium) containing 10% foetal calf serum (GIBCO). Fifty $\mu$l of dilutions of crude supernatant or S-300 chromatographic fractions were added in triplicate to the cells. The plates were incubated for an 20 h, at 37°C in a 5% $CO_2$ incubator. Then, 10 $\mu$l of a 3-4,5-dimethylthiazol-2-yl-3,5-diphenylformazan solution (5 mg $ml^{-1}$ PBS) (MTT, Sigma) were added to each well and the plates incubated for an additional 4 h. After that, 100 $\mu$l of isopropanol containing 0.04 N HCl were added to each well. Fifteen min later, the degree of cell lysis was quantitated spectrophotometrically (630 nm) by using an enzyme-linked immunoassay analyser (Multiskan MCC/340 MKII, Flow Laboratories). Because of the unavailability of rat TNF, standard curves were done with mrTNF-$\alpha$ (Genentech Inc.) and TNF content in assayed materials were calculated by comparison with the standard amounts. The results correspond to the means of data obtained in at least three different experiments. In some experiments, we use a rabbit anti-mrTNF antibody to study the involvement of TNF-like cytokine in our samples.

*Measurement of rat paw oedema:* Oedema was measured plethysmographically.[21] The volume increase ($\Delta$ volume) of the inflamed paw was obtained by comparing the observed volume before and after the intra-plantar (i.pl.) administration of 100 $\mu$g/0.1 ml of carrageenin, dextran (Dextran 70, Pharmacia) or OVA. Animals were pretreated with either sterile PBS or P2 chromatographic fraction 15 min before i.pl. challenge. Oedema was measured 2, 4 or 6 h after i.pl. challenge, except when indicated.

### Results

*Neutrophil migration activities of S-300 chromatographic fractions:* The '*in vivo*' inhibitory activity displayed by crude NRIF was detected in fraction P2 of S-300 chromatography of macrophage supernatants (Fig. 1, Panel A). The elution volume of P2 corresponded to an apparent molecular weight of 500 KDa.

When tested for TNF activity, crude supernatant was demonstrated to contain significant amounts (500 ng/0.2 ml), but only small amounts of such cytokine (1 pg $ml^{-1}$) were detected in P2 fraction. Antibodies raised against mouse recombinant TNF-$\alpha$ abolished the cytotoxicity of the mrTNF-$\alpha$ and strongly reduced the activity of the crude supernatant (50–60% of inhibition). We have tested increasing doses of mrTNF-$\alpha$ (0.3 pg–3 $\mu$g) upon carrageenin-induced neutrophil migration. Only TNF-$\alpha$ doses $10^6$ times greater than that found in P2 were able to significantly inhibit neutrophil migration to rat peritoneal cavities (data not shown).

In panel B of Fig. 1, it is shown that P2 fraction of S-300 chromatography did not inhibit '*in vitro*' chemotaxis induced by FMLP, while it was found significant inhibition in fractions 5 to 10.

When chromatographic fractions were tested for MNCF, the highest activity was eluted in the void volume (P1) and in a fraction corresponding to 30–60 KDa (P7) (data not shown). In addition, we have separately studied the effect of P2 injected into peritoneal cavities. Figure 2 shows that P2 caused neutrophil migration (left block of bars) that was significantly reduced by pretreatment of the animals with dexamethasone (right block of bars).

Besides its inhibitory effect upon neutrophil migration induced by carrageenin, P2 fraction also inhibited neutrophil recruitment induced by either LPS or FMLP into rat peritoneal cavities (Fig. 3).

*Effect of P2 fraction upon oedema:* Figure 4 shows that P2 fraction inhibited carrageenin (left group of bars) but not the oedema induced by dextran (right group of bars), as measured 2 h after inflammatory stimuli. The Fig. 5 shows that P2 fraction injected before ovalbumin challenge reduced the oedema (full and open circles) but did not affect the paw volume of control animals previously treated with CFA only (open and full squares).

### Discussion

Previous work from our laboratory have indicated the presence of a neutrophil recruitment inhibitory activity in thioglycollate-elicited and

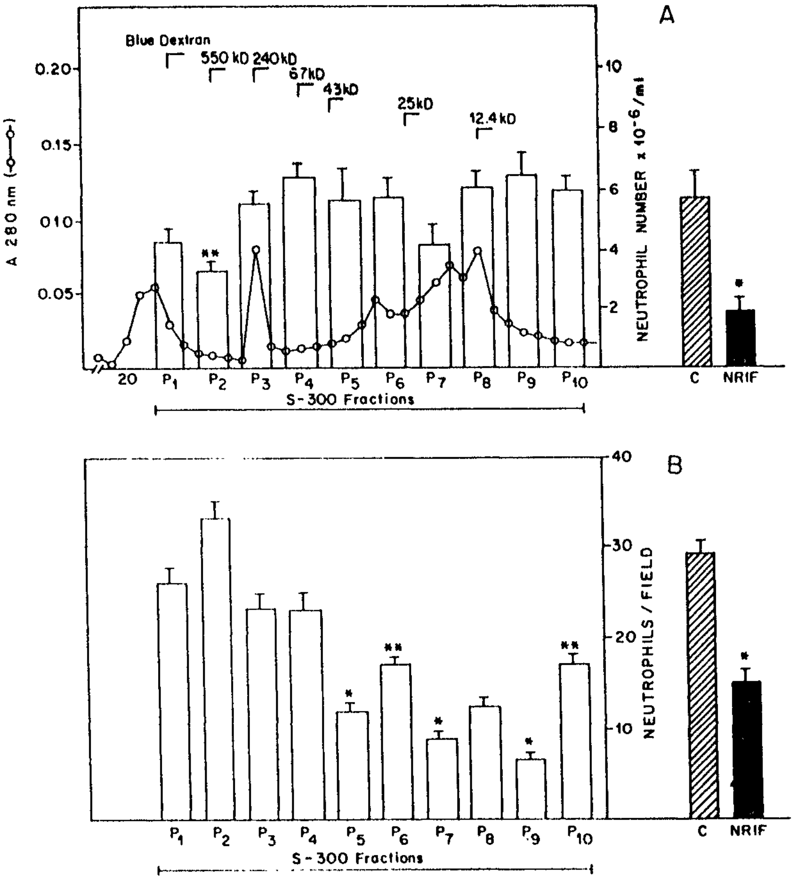

FIG. 1. Effect of S-300 chromatographic fractions on neutrophil migration and chemotaxis. Panel A shows neutrophil migrations induced by i.p. injection of carrageenin (300 $\mu$g/rat) in animals pretreated 15 min before with i.v. injections of PBS (C, control), S-300 chromatographic fractions (P1–P10) or crude supernatant (NRIF), as indicated in Material and Methods. Traced line (-O-O-) represents the absorbance of each fraction (280 nm), starting with fraction 20. The results are reported as means ± SEM of six animals per experimental group.* $p < 0.01$ and **$p < 0.05$ as compared to control group. Panel B shows the effect of the pretreatment of human neutrophils with S-300 fractions on chemotaxis induced by FMLP ($5 \times 10^{-8}$ M). The results are expressed as means ± SEM of cells counted in 15 microscopic fields. *$p < 0.01$ and **$p < 0.05$ as compared to control group. Student's *t*-test.

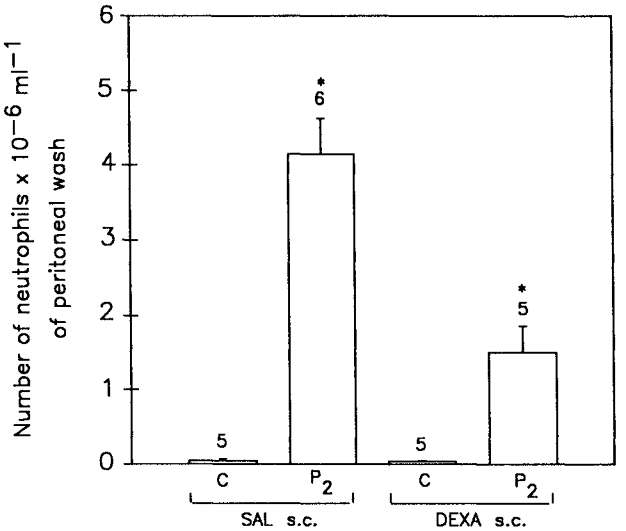

FIG. 2. Detection of MNCF activity in P2 fraction. The bars represent neutrophil migration into peritoneal cavities induced by either saline (C, control) or P2 fraction, in either saline or dexamethasone (0.5 mg kg$^{-1}$, s.c., 1 h before) pretreated animals. The results are reported as means ± SEM of the number of animals indicated up each bar. *$p < 0.01$ as compared to control. Student's *t*-test.

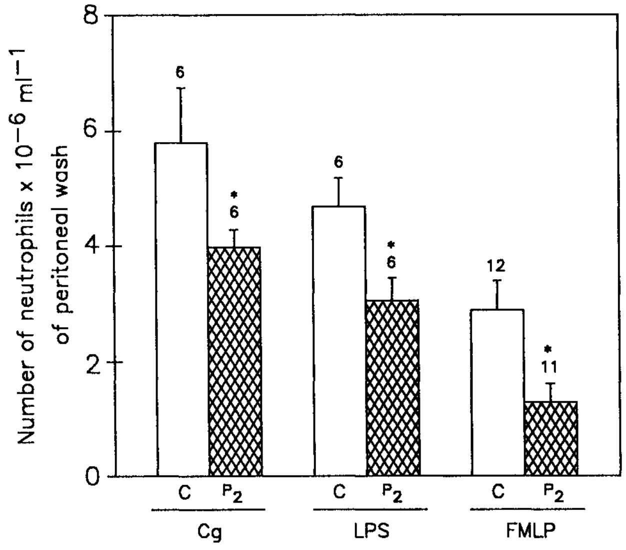

FIG. 3. Inhibitory effect of P2 fraction upon neutrophil migration induced by different inflammatory stimuli. Bars represent neutrophil migrations induced by i.p. injections of carrageenin (Cg, 300 $\mu$g), LPS (5 ng) or FMLP (130 ng), in animals pretreated with i.v. injection of P2 fraction. The results are reported as means ± SEM of the number of animals indicated up each bar. *$p < 0.05$ as compared to control group. Student's *t*-test.

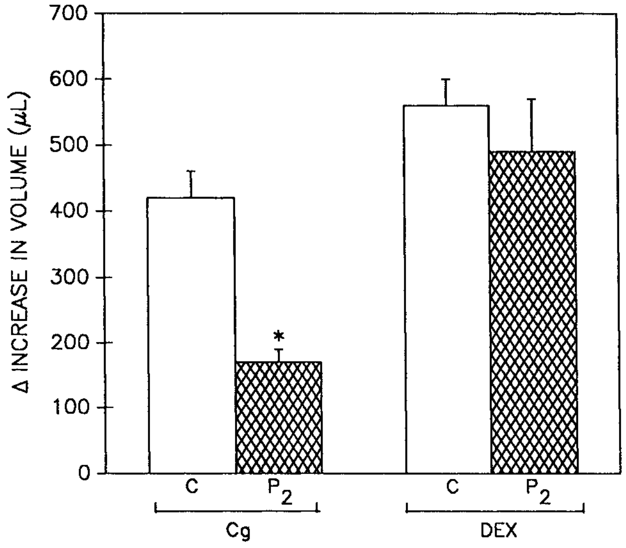

FIG. 4. Inhibitory effect of P2 fraction on carrageenin-induced oedema. The bars show the increase in volume induced by 100 μg i.pl. injection of either carrageenin (Cg) or dextran (DEX) in animals pretreated 15 min before with i.v. injection of either PBS: (C, control) or P2 fraction (P2). Oedema was measured 4 h after challenge. The results are reported as the means ± SEM of five animals per experimental group. $^*p < 0.01$ as compared to controls. Student's *t*-test.

LPS-stimulated macrophage supernatants.[18] This activity was referred to as neutrophil recruitment inhibitory factor (NRIF), since it was specific for polymorphonuclear leucocytes and in contrast with other substances, such as LPS, it did not inhibit mononuclear cell migration to rat peritoneal cavities.[22] This result indicates that NRIF is not contaminated with LPS. Further evidence that NRIF activity is not due to LPS contamination is based upon the fact that polymyxin B did not affect NRIF activity but abolished LPS neutrophil

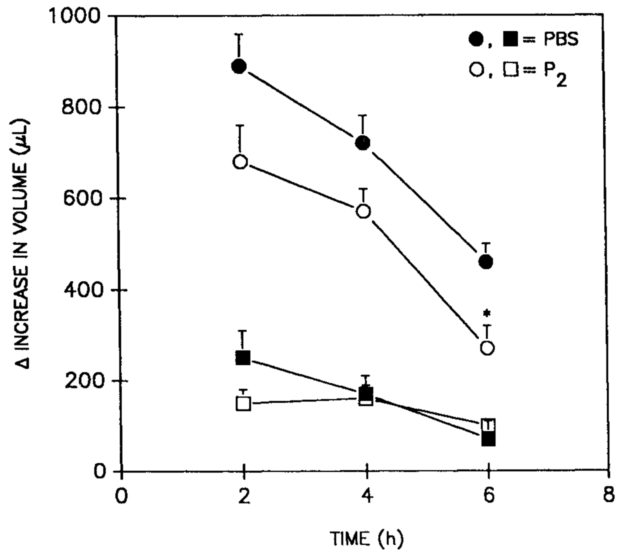

FIG. 5. Inhibitory effect of P2 fraction on immunologically-induced oedema. Immunization procedure was performed with OVA + CFA (circles) or with CFA only (squares). On day of experiment, rats were pretreated 15 min before with i.v. injection of either PBS (C, control) or P2 fraction (P2) and challenged with 100 μg i.pl. injection of OVA. Oedema was measured 2, 4 and 6 h. The results are expressed as indicated in Fig. 4. $^*p < 0.05$ as compared to control. Student's *t*-test.

recruitment inhibitory effect.[18] Furthermore, resident macrophage monolayers release the same inhibitory activity without LPS stimulation.[23] In the present study we showed that NRIF activity was present in a fraction, referred as P2, obtained by gel filtration chromatography of the crude macrophage supernatant on Sephacryl S-300. This fraction was eluted in a volume corresponding to high molecular weight proteins (240–550 KDa) as deduced by standard calibration curve.

P2 fraction was effective against other inflammatory stimuli than carrageenin, such as LPS or FMLP, the latter being a direct chemotactic attractant[24] in '*in vivo*' inhibitory assays. In contrast, P2 fraction did not inhibit neutrophil chemotaxis induced by FMLP in microchemotaxis assay. These results taken together, suggest that NRIF did not affect the ability of neutrophils to respond to chemotactic stimuli, thus supporting the suggestion that NRIF may be acting by blocking neutrophil-endothelial adhesion mechanisms.[18] However, additional experiments with more purified material are required to clarify its precise mechanism of action.

Similar to NRIF, i.v. administration of TNF-α has been shown to inhibit neutrophil accumulation at the site of inflammatory stimulus injection.[6] Our results showed that crude macrophage supernatants contain significant TNF amounts (blocked by rabbit anti-mrTNF antibodies) that may contribute to inhibitory activity displayed by this material. However, P2 fraction presented only traces of TNF, corresponding to a quantity $10^6$ times slower than that necessary to present neutrophil recruitment inhibitory activity. Circumstantial evidence that NRIF inhibitory activity is not due to TNF is supported by the high apparent molecular weight of the P2 fraction.

IL-8 is another cytokine presently shown to inhibit neutrophil accumulation at sites of acute inflammation when administered intravenously.[7] The possibility that NRIF activity may be contaminated by IL-8 is minimized by its high molecular weight elution volume and by the evidence that no traces of IL-8 was found in a sample of P2 fraction (ELISA, using a monoclonal antibody raised against human recombinant IL-8, sensitivity of detection smaller than 50 pg, see acknowledgements). It should be pointed out, however, that the absence of detection could be due to the low sensitivity of the test to rat IL-8. However, in an on going experiment in our laboratory, it was found that antibodies raised against human IL-8 are able to inhibit either carrageenin as well as hIL-8-induced hyperalgesia in the rat.

Previous results obtained in our laboratory showed the presence of IL-1 in crude supernatant of LPS-stimulated macrophages.[18] However, as demonstrated by the authors, IL-1 does not block neutrophil migration to peritoneal cavities even if it is injected in doses higher than those detected in such supernatants.

Another inflammatory parameter, rat paw oedema induced by either carrageenin or antigen challenge, was significantly reduced by P2 fraction administration. This effect was not observed with dextran-induced oedema. Carrageenin and dextrans have been shown to induce increased vascular permeability by different mechanisms. While dextrans induce fluid accumulation due to mast cell degranulation with little protein and few neutrophils, carrageenin induces a protein-rich exudate containing large number of neutrophils.[25] Since NRIF did not present inhibitory activity upon dextran-induced oedema, we suggest that the factor only acts in polymorphonuclear-leucocyte dependent inflammatory responses. Thus, it is plausible that P2 is blocking immunologically-induced oedema by a similar mechanism. If this hypothesis is correct, we can envisage its usefulness in the control of immunological diseases such as bronchial asthma, where infiltration of inflammatory cells has been related to bronchial hyperresponsiveness.[26] Further evidence that NRIF activity is not due to IL-1 or TNF presence has been demonstrated by the results that i.v. injected rh-IL1 ($\alpha$ and $\beta$) and TNF-$\alpha$ inhibit both carrageenin and dextran-induced oedema.[27]

Several cytokines, including TNF-$\alpha$ and $\beta$, IL1-$\alpha$ and $\beta$,[15] gamma interferon[16] and IL-8[17,28] are reported to induce neutrophil migration. In contrast to MNCF, dexamethasone inhibited the activity of those known cytokines.[15–17] We have observed that '*in vivo*' chemotactic activity displayed by P2 was significantly blocked by pretreatment of the animals with dexamethasone, thus indicating that there is a material which indirectly stimulates neutrophil emigration. This activity could result from the presence of traces of several cytokines or due to NRIF by itself. The residual emigration induced by P2 in rats pretreated with dexamethasone may result from the presence of a direct acting cytokine, such as MNCF since this activity was eluted in two peaks, one of them corresponding to the void volume fraction, eluted just before P2. Further studies are necessary to clarify this point.

In the light of the present results it is possible to assume NRIF as a candidate for a novel cytokine, capable of inhibiting neutrophil migration to an inflammatory site when present in the circulation, as might occur in endotoxemic shock.

### References

1. Cybulsky MI, Chan MK, Movat HZ. Acute inflammation and microthrombosis induced by endotoxin, IL-1, and TNF and their implication in gram-negative infection. *Lab Invest* 1988; **58:** 365-378.
2. Opp M, Obal F Jr, Cady AB, *et al.* Interleukin-6 is pyrogenic but not somnogenic. *Physiol Behavior* 1989; **45:** 1069–1072.
3. Cannon JG, Tompkins RG, Gelfand JA, *et al.* Circulating interleukin-1 and tumor necrosis factor in septic shock and experimental endotoxin fever. *J Inf Dis* 1990: **161:** 79–84.
4. Van Zee KJ, Deforge LE, Fischer E, *et al.* IL-8 in septic shock, endotoxemia and after IL-1 administration. *J Immunol* 1991; **146:** 3478–3482.
5. Gimbrone MA, Obin MS, Brock AF, *et al.* Endothelial interleukin-8: a novel inhibitor of leucocyte-endothelial interactions. *Science* 1989; **246:** 1601–1603.
6. Otsuka Y, Nagano K, Hori K, *et al.* Inhibition of neutrophil migration by tumor necrosis factor. *Ex vivo* and *in vivo* studies in comparison with *in vitro* effect. *J Immunol* 1990; **145:** 2639–2643.
7. Hechtman DH, Cybulsky MI, Fuchs HJ, *et al.* Intravascular IL-8. Inhibitor of polymorphonuclear leucocyte accumulation at sites of acute inflammation. *J Immunol* 1991; 147: 883–892.
8. Ulich TR, Yin S, Guo K, *et al.* Intratracheal injection of endotoxin and cytokines. II. Interleukin-6 and transforming growth factor beta inhibit acute inflammation. *Am J Pathol* 1991; **138:** 1097–1101.
9. Yoshimura T, Matsushima K, Oppenheim JJ *et al.* Neutrophil chemotactic factor produced by lipopolysaccharide (LPS)-stimulated human blood mononuclear leucocytes: partial characterization and separation from interleukin-1 (IL-1). *J Immunol* 1987; **139:** 788–793.
10. Walz A, Peveri P, Aschauer H, *et al.* Purification and amino acid sequencing of NAF, a novel neutrophil-activating factor produced by monocytes. *Biochem Biophys Res Commun* 1987; **149:** 755–762.
11. Cicco NA, Lindemann A, Content J, *et al.* Inducible production of IL-6 by human polymorphonuclear neutrophils: Role of GM-CSF and TNF. *Blood* 1990; **75:** 2049–2052.
12. Gregory H, Young J, Schroder JN, *et al.* Structure determination of a human lymphocyte derived neutrophil activating peptide (LYNAP). *Biochem Biophys Res Commun* 1988; **151:** 883–890.
13. Strober W, James SP. The interleukins. *Pediatr Res* 1988; **24:** 549–557.
14. Cunha FQ, Ferreira SH. The release of a neutrophil chemotactic factor from peritoneal macrophages by endotoxin: inhibition by glucocorticoids. *Eur J Pharmacol* 1986: **129:** 65–76.
15. Faccioli LH, Souza GEP, Cunha FQ, *et al.* Recombinant interleukin-1 and tumor necrosis factor induce neutrophil migration '*in vivo*' by indirect mechanisms. *Agents & Actions* 1990; **30:** 344–349.
16. Ribeiro RA, Cunha FQ, Ferreira SH. Recombinant gamma interferon causes neutrophil migration mediated by the release of a macrophage neutrophil chemotactic factor. *Int J Exp Pathol* 1990; **71:** 717–725.
17. Ribeiro RA, Flores CA, Cunha FQ, *et al.* IL-8 causes *in vivo* neutrophil migration by a cell dependent mechanism. *Immunol* 1991; **73:** 472–477.
18. Cunha FQ, Souza GEP, Souza CAM, *et al. In vivo* blockage of neutrophil migration by LPS is mimicked by a factor released from LPS-stimulated macrophages. *Br J Exp Pathol* 1989; **70:** 1–8.
19. Souza GEP, Ferreira SH. Blockade by antimacrophage serum of the migration of PMN neutrophils into the inflamed peritoneal cavity. *Agents & Actions* 1985; **17:** 97–103.
20. Spevik T, Nissen-Meyer J. A highly sensitive cell line, WEHI 164 clone 13, for measuring cytotoxic factor/tumor necrosis factor from human monocytes. *J Immunol Methods* 1986; **95:** 99–105.
21. Ferreira SH. A new method for measuring variations of rat paw volume. *J Pharm Pharmacol* 1979; **31:** 648.
22. Tavares BM, Cunha FQ, Ferreira SH. Macrophages stimulated with lipopolysaccharide release a selective neutrophil recruitment inhibitory factor: an *in vivo* demonstration. *Braz J Med Biol Res* 1989; **22:** 733–736.
23. Tavares-Murta, BM. Inhibition of neutrophil migration by a factor released from LPS-stimulated macrophages. Thesis (Master Degree). Faculty of Medicine of Ribeirão Preto, University of Sao Paulo, 1991.
24. Cybulsky MI, McComb DI, Movat HZ. Protein synthesis dependent and independent mechanisms of neutrophil emigration. Different mechanisms of inflammation in rabbits induced by interleukin-1, tumor necrosis factor alpha or endotoxin versus leucocyte chemoattractants. *Am J Pathol* 1989; **135:** 227–237.
25. Lo TN, Almeida AP, Beaven MA. Dextran and carrageenan evoke different inflammatory responses in rat with respect to composition of infiltrates and effect of indomethacin. *J Pharmacol Exp Ther* 1982; **221:** 261–267.
26. Macklem PT. A hypothesis linking bronchial hyperreactivity and airway inflammation: implications for therapy. *Ann Allergy* 1990; **64:** 113–116.
27. Nakamura H, Motoyoshi S, Kadokawa T. Anti-inflammatory action of interleukin-1 through the pituitary-adrenal axis in rats. *Eur J Pharmacol* 1988; **151:** 67–73.
28. Colditz I, Zwahlen R, Dewald B, *et al. In vivo* inflammatory activity of neutrophil-activating factor, a novel chemotactic peptide derived from human monocytes. *Am J Pathol* 1989 **134:** 755–760.

ACKNOWLEDGEMENTS. We thank Neomesia IS Freiri, Ieda R dos Santos, Sandra MO Thomaz and A Castania Filho for technical assistance. We thank Genentech Assay Services for quantifying IL-8 in P2 sample. WMSC Tamashiro is grateful to UNICAMP for granting her a leave of absence to conclude her postdoctoral research. RMD Nogueira is grateful to Universidade Federal do Ceara for granting her a leave of absence to conclude her PhD research. WMSC Tamashiro and BM Tavares-Murta also acknowledge the support their study received from CNPq. Research supported by FAPESP and CNPq.

Received 20 November 1991;
accepted in revised form 23 December 1991
